# Vasectomy and prostate cancer risk: a meta-analysis of cohort studies

**DOI:** 10.1038/srep09920

**Published:** 2015-04-30

**Authors:** Yonggang Shang, Guangwei Han, Jia Li, Jiang Zhao, Dong Cui, Chengcheng Liu, Shanhong Yi

**Affiliations:** 1 Department of Urology, Xinqiao Hospital, Third Military Medical University, Chongqing, 400037, China; 2 Department of Urology, The 169th hospital of People's Liberation Army, Hengyang, Hunan Province, 421002, China

## Abstract

Some studies have suggested that vasectomy is associated with the increased risk of prostate cancer, however, this conclusion is not supported by all the published studies. In order to examine the relationship between vasectomy and prostate cancer risk, we conducted a meta-analysis of cohort studies to clarify this controversial association. PubMed and Medline were used to identify the cohort studies that reported the association of vasectomy with prostate cancer risk from 1980 to January 2015. Based on a random effects model, the RR and 95% CI were used to assess the combined risk. In total, 10 cohort studies involving more than 7027 cases and 429914 participants were included. There was no significant relationship between vasectomy and prostate cancer risk, the pooled RR (95%CI) was 1.11[0.98, 1.27] (P = 0.109). In subgroup-analysis, the relationship between vasectomy and prostate cancer risk was not significantly modified by the length of follow-up and population distribution except Americans. Omission of any single study had little effect on the pooled risk estimate. Little evidence of publication bias was found. In conclusion, our meta-analysis suggests that vasectomy is not associated with the increased risk of prostate cancer. More studies based on other populations including the Chinese are needed.

Vasectomy is a common and reliable way of permanent contraception among the adult men^1^. In the USA, approximately 15% males undergo this surgical operation^2^. As a basic state policy for China, it plays an important role in the family planning. However, some studies have reported that vasectomy is linked to the increased risk of prostate cancer^3^,^4^. These negative results are bound to influence the clinical application of vasectomy.

It has seen a rapidly increasing interest in the relationship between vasectomy and the risk of prostate cancer in the past two decades. Many studies have examined this relationship^5^,^6^,^7^,^8^,^9^,^10^, some reported a positive association between vasectomy and prostate cancer risk^5^,^7^,^8^, however, others found a null association^6^,^9^,^10^. Although some previous studies had clarified the association between vasectomy and prostate cancer risk, the results were still inconsistent^11^,^12^. Moreover, the evidence was limited because case-control studies and only a few cohort studies were included, which would lead to more confound factors and biases. In addition, some cohort studies^13^,^14^ were not included in the previous studies and some newly emerging cohort studies have observed this association recently^15^,^16^. With the accumulating evidences, we conducted an update meta-analysis of cohort studies to improve understanding and re-estimate the association between vasectomy and prostate cancer risk.

## Methods

### Study retrieval and selection

PubMed and Medline were used to identify the studies that reported the relationship between vasectomy and the risk of prostate cancer which were published from 1980 to January 2015. The following keywords were used: “vasectomy” in combination with “prostate cancer” and “prostate carcinoma”. The reference lists of the retrieved studies were reviewed. If necessary, we contacted the authors of the original studies for the required data.

The studies that met the following criteria could be included: 1. the study had a cohort study design; 2. the study reported the association between vasectomy and the risk of prostate cancer; 3. the publication language was confined to English; 4. if there were duplicate publications on the same study population or authors, we included the most recent one. Firstly, we checked the titles and abstracts to confirm the potential studies. If uncertain, a subsequent full-text assessment was conducted. The study retrieval was conducted by two independent authors (Shang Y.G. and Han G.W.). All disputes were resolved by discussion.

### Data extraction and quality assessment

We conducted the data extraction with a standardized form. The following information was collected from the included studies: the last name of the first author, publication year, study population, number of cases and participants, length of follow-up, estimated effects from the most fully adjusted model and the corresponding 95% CI for the relationship between vasectomy and prostate cancer risk.

Rather than reporting the aggregate scores, the key components of designs, such as selection of study populations, ascertainment of exposure and outcome, duration of follow-up, were used to estimated the quality of primary studies.

### Statistical analysis

All statistical analyses were conducted with STATA version 12.0. The pooled RR and the corresponding 95%CI were calculated to assess the relationship between vasectomy and prostate cancer risk by using the assumptions of a random-effects model that considered within-study and between-study variation^17^. We investigated the heterogeneity in results across the included studies by using the Q and I^2^ statistics^18^. A subgroup analysis was performed to identify the source of heterogeneity, if possible, and the effect of the potential factors on the overall risk estimate. In addition, we conducted a sensitivity analysis to investigate the influence of a single study on the overall risk estimate by omitting one study at a time. Begg's and Egger's test were used to detect the evidence of a publication bias. In our study, if the P-value was less than 0.05, it was considered statistically significant.

## Results

### Study selection process

**Figure 1:**
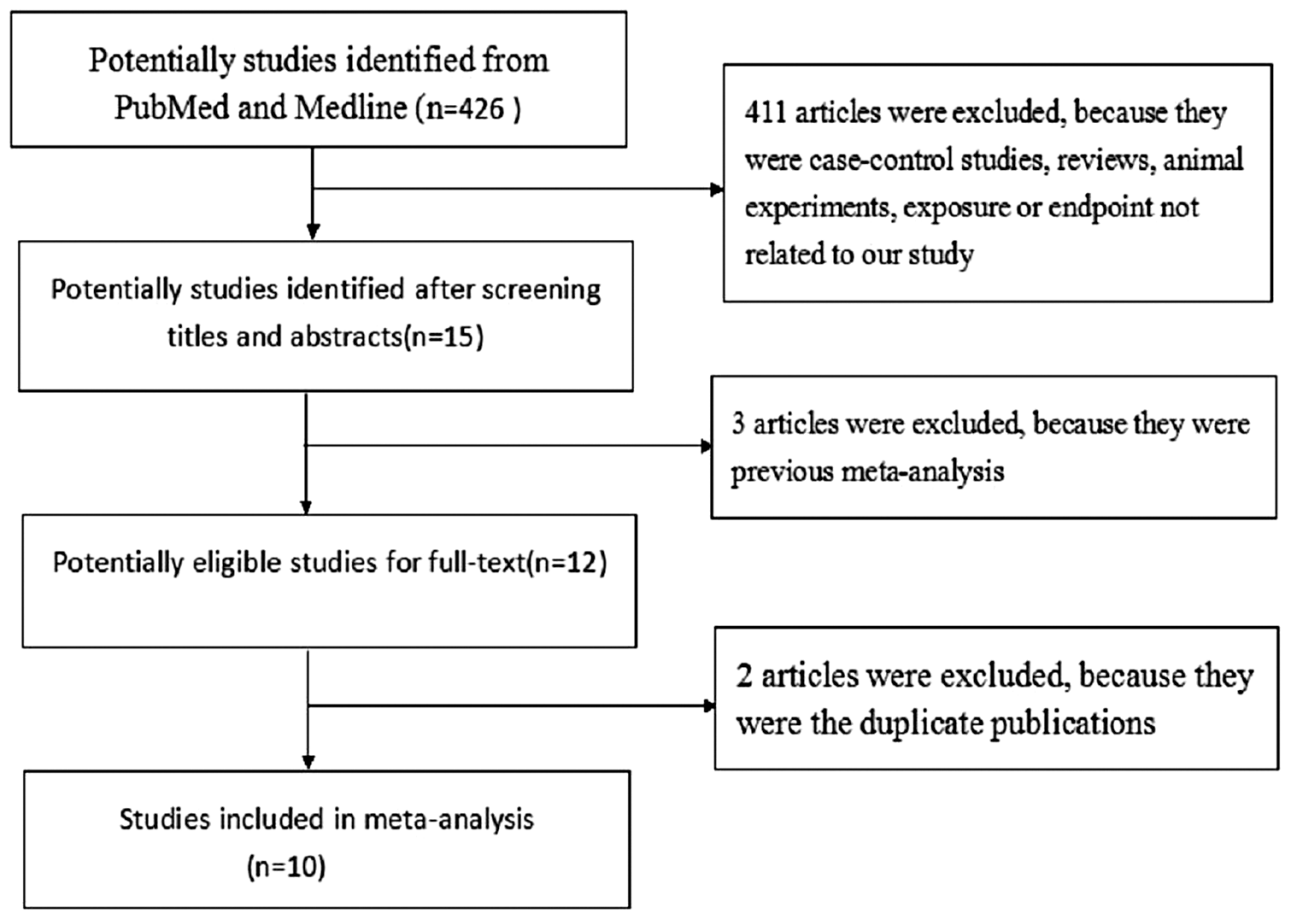
Flow chart of study selection.

Figure 1 presents the flow chart of articles selection process. PubMed and Medline were used to identify relevant studies. We initially identified 426 potential studies from the two databases (201 were from PubMed and 225 were from Medline). After screening the abstracts or titles, most of them were excluded, because they were case-control studies, letters and reviews, or the exposure and endpoint were not linked to our study or they were duplicate publications. After that, 10 cohort studies involving more than 7027 cases and 429914 participants were included^5^,^6^,^8^,^13^,^14^,^15^,^16^,^19^,^20^,^21^.

### Characteristics of the included studies

The main characteristics of included 10 cohort studies are presented in Table 1. The publication years of them ranged 1991 to 2014. Of them, 6 were conducted among American males^5^,^8^,^13^,^14^,^16^,^19^, 1 in England^20^ and Brazil^15^, 2 in Denmark^6^,^21^, respectively. 2 studies were based on all years old populations^6^,^19^, 6 studies were conducted among the middle-aged and elderly populations^5^,^8^,^14^,^15^,^16^,^20^, but the other 2 studies did not report this^13^,^21^. The length of follow-up was from 2 to 24 years. All studies included a large sample size.

### Data analysis

**Figure 2:**
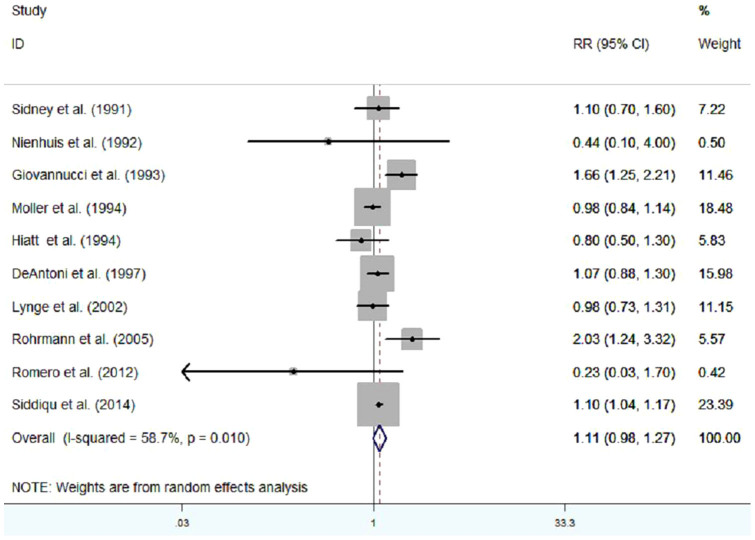
Meta-analysis of cohort studies on vasectomy and risk of prostate cancer.

The RRs from the 10 cohort studies and pooled RR are presented in Figure 2. In total, 10 included studies were used to assess the association between vasectomy and prostate cancer risk. Of them, 3 showed that vasectomy was linked to an increased risk of prostate cancer^5^,^8^,^16^. Overall, our study suggested that there was no statistically significant relationship between vasectomy and prostate cancer risk, the pooled RR (95%CI) was 1.11[0.98, 1.27] (P = 0.109). But we observed evidence of heterogeneity (I^2^ = 57.8%, P < 0.05).

**Figure 3:**
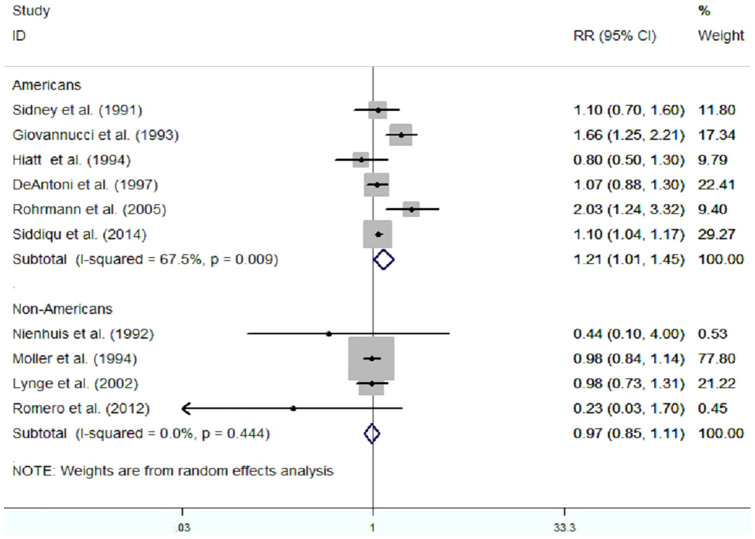
Subgroup-analysis of observational studies on vasectomy and risk of prostate cancer according to population distribution.

Meanwhile, we conducted a subgroup-analysis to identify the effect of population distribution on the pooled risk estimate. The results of this subgroup-analysis is presented in Figure 3. We found that there was a significant positive relationship between vasectomy and prostate cancer risk among American males (RR(95%CI) was 1.21 [1.01, 1.45], p = 0.044), but this positive association was not found in the non-American males (RR(95%CI) was 0.97[0.85,1.11], p = 0.653).

**Figure 4:**
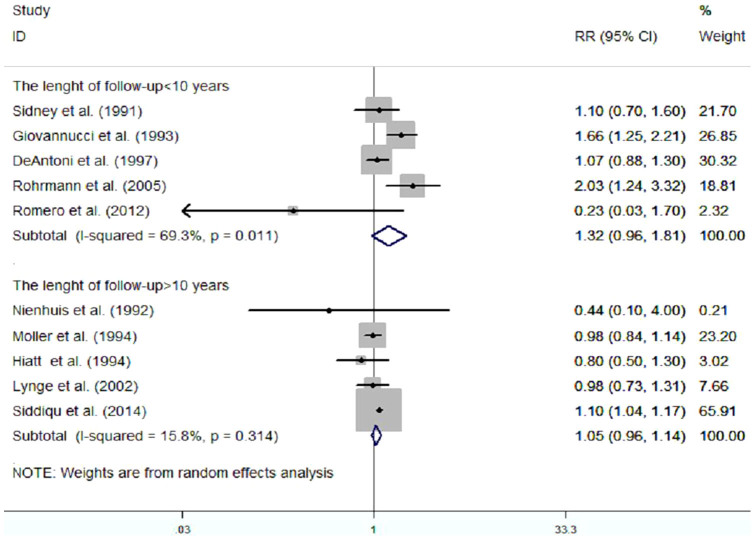
Subgroup-analysis of cohort studies on vasectomy and risk of prostate cancer according to the length of follow-up.

In addition, we assessed the effect of the length of follow-up on the pooled RR by conducting another subgroup-analysis (Figure 4). We did not identify the association between vasectomy and the risk of prostate cancer in the groups in which both the length of follow-up was less than and more than 10 years, the RR(95%CI) was 1.32[0.96,1.81](p = 0.089), 1.05[0.96, 1.14] (p = 0.263), respectively.

**Figure 5:**
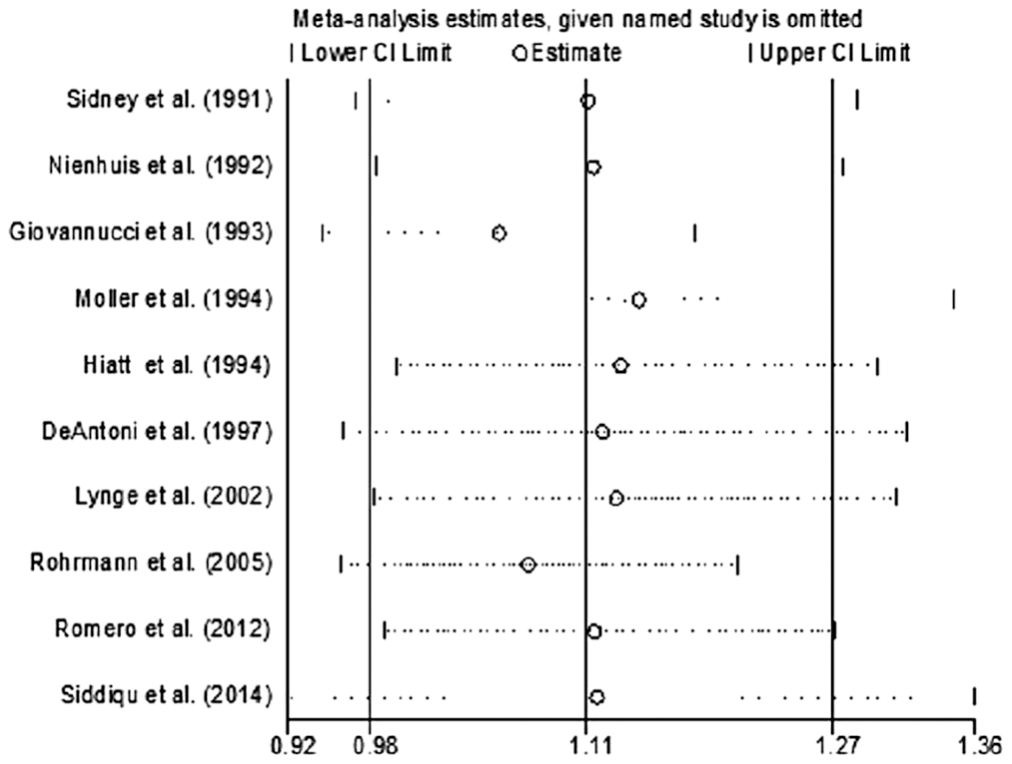
Forest plot for sensitivity analysis.

By removing one study at a time, we conducted a sensitivity analysis to assess the influence of each included study on the pooled RR. The combined RRs were similar to each another, and none significantly modified the pooled RR. But after excluding 2 studies^5^,^8^, there was no evidence of heterogeneity among the remaining studies (I^2^ = 0.1%, P = 0.428), which suggested that these 2 studies were the main source of heterogeneity. The result of sensitivity analysis is presented in Figure 5.

**Figure 6:**
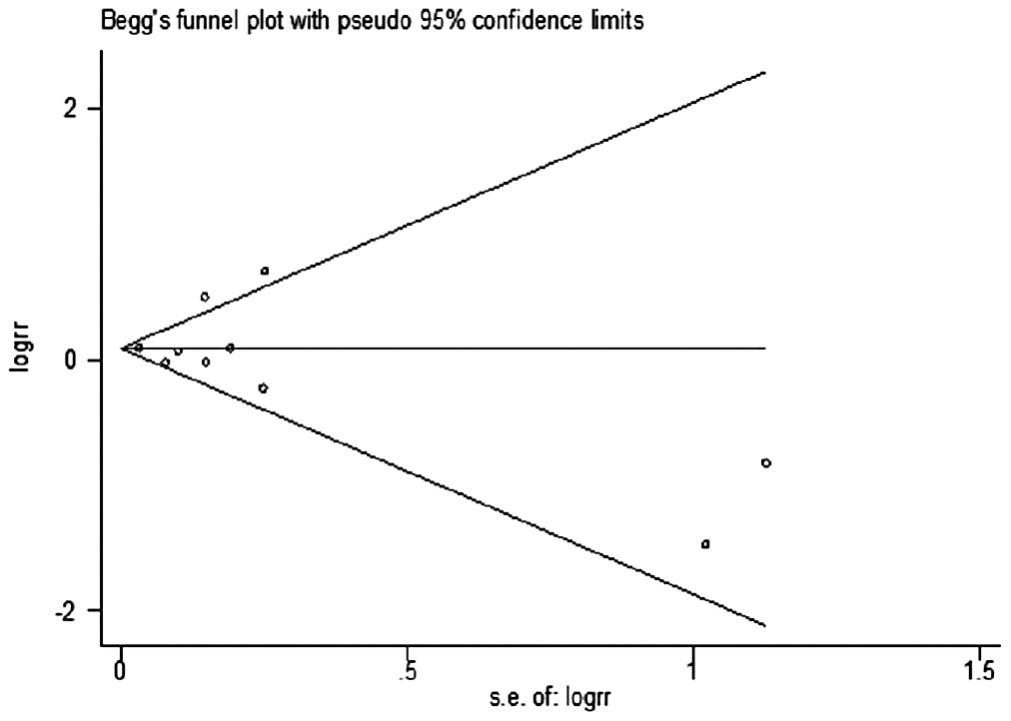
Forest plot for publication bias.

The funnel plot showed all the included studies symmetrically distributed in the triangle area. Begg's and Egger's regression test showed a low probability of publication bias in our study (P = 0.878). The funnel plot of the studies is presented in Figure 6.

## Discussion

Our meta-analysis suggests that vasectomy is not associated with the increased risk of prostate cancer. All included studies had a cohort study design, however, there was substantial evidence of heterogeneity, the different populations, varied characteristics of participants and length of follow-up might partially explain it. We searched the source of heterogeneity by conducting a sensitivity analysis and we found that 2 studies were the main source of heterogeneity^5^,^8^. These 2 studies were similar to most included studies, for instance, they were based on American males, however, these two studies had a prospective cohort study design and which reported a significant positive relationship between vasectomy and prostate cancer risk.

The subgroup-analysis showed that the relationship between vasectomy and prostate cancer risk was not significantly modified by the length of follow-up and population distribution except American males. This result was not wondrous, because 6 studies^5^,^8^,^13^,^14^,^16^,^19^ including the 3 studies^5^,^8^,^16^ which found a significant positive relationship between vasectomy and prostate cancer risk were conducted among American men, however, the other studies which were based on the non-American males did not report similar positive results.

Vasectomy is a common method of male sterilization, whether it is linked to the increased risk of prostate cancer has been debated for many years. In 1980s, many case-control studies showed that vasectomy history was not linked to the increased risk of prostate cancer^22^,^23^, however, the following case-control studies suggested inconsistent results^24^,^25^,^26^. The selection and recall bias and smaller sample sizes of these case-control studies might limit the statistical effect and explained the varied findings. In order to get more reliable results, cohort studies were indispensable. To our knowledge, the Kaiser Permanente study which was conducted in 1987 was the first one to estimate the relationship between vasectomy and prostate cancer risk by using a cohort study design^27^, this study also showed that vasectomy did not increase the risk of prostate cancer. In 1991, a second Kaiser Permanente study showed a similar result^19^. However, in 1993, *Giovannucci et* *al* found a significant positive association of vasectomy with prostate cancer risk in American males^5^, however, in this study, the association between vasectomy and prostate cancer observed in epidemiological study is highly unlikely owing to chance, the potential influences of confounding and bias must be considered seriously. In the past two decades, only 2 studies reported that there was an increased risk of prostate cancer in males with vasectomy^8^,^16^, one showed that vasectomy was associated with a modest increased risk of prostate cancer^16^ and another one showed a strong positive relationship association between vasectomy and the risk of prostate cancer^8^. They pointed out that the biologic mechanisms of the association between vasectomy and prostate cancer were unclear, physiologic changes in men after vasectomy included local effects on the testis and effects that had potential systemic implications^28^. These physiologic changes might ultimately lead to the development of prostate cancer^29^. However, these two studies were still based on American males and men who underwent a vasectomy might be more likely to return to hospitals for prostate cancer screening than men who did not underwent this sterilizing operation. Therefore, they found a positive between vasectomy and the risk of prostate cancer. However, the others did not find a positive association. Although these cohort studies enhanced the statistical power and expanded the sample size effectively, the results were inconsistent. Facing of the inconsistent results, we conducted a meta-analysis and indicated that the patients with vasectomy did not had an increased risk of prostate cancer.

The present meta-analysis had some strengths. First, it was the updated one to assess the relationship between vasectomy and prostate cancer risk by combining cohort studies. We knew that in case-control studies, artificial association might appear due to inadequate selection of controls^30^, but in our study, the included 10 cohort studies significantly reduced the recall and selection biases. Second, our study included more than 7027 cases and 429914 participants that greatly enhanced the statistical power and provided more reliable results. Above all, we found that vasectomy was not associated with the increased risk of prostate cancer, the decision to opt for a vasectomy brought benefits to public health.

Meanwhile, some limitations should be considered. First, residual confounders and unmeasured factors always consist in observational studies. Residual confounders or unmeasured factors are still possible after adjusting for the most relevant confounding factors^31^. For example, men with a long-time regular aspirin use have a 14% decreased risk of prostate cancer^32^, if the participants are suffered from coronary disease, the long-time regular aspirin use is inevitable, which may influence the risk estimate. Second, there was strong evidence of heterogeneity among the included studies. Although we had detected the major source of heterogeneity by conducting sensitivity analysis, other differences through the studies should be considered. In 2 studies^6^,^19^, the range of age was all years old, but other studies only included the elderly. It is well known that the incidence of prostate cancer among the elderly is significantly higher than that in the younger, hence, the overall incidence of prostate cancer may be lower in these 2 studies. Third, vasectomy is a common way of permanent contraception among the Chinese males, however, our findings were mainly based on the western populations, especially American men, more studies based on other populations are necessary to verify the result.

## Conclusions

Our meta-analysis suggests that vasectomy is not associated with the increased risk of prostate cancer. More studies based on other populations including the Chinese are warranted.

## Author Contributions

S.Y.G., C.D., H.G.W. and L.J. wrote and revised the main manuscript text; Z.J., L.C.C. and Y.S.H. prepared all the figures. All authors reviewed the manuscript.

## Figures and Tables

**Table 1:** The characteristics of included studies

Study (year)	Country	Range of age	Sample Size(n) case/participant	Adjusted RR (95% CI)	Years of follow-up
**Sidney et al.** (1991)	USA	all years	135/20476	1.0[0.7, 1.6]	5
**Nienhuis et al.** (1992)	England	25–49	5/35446	0.44[0.1,4.0]	16
**Giovannucci et al.** (1193)	USA	40–75	279/47855	1.66[1.25, 2.21]	4
**Moller et al.** (1994)	Denmark	N*	165/73 917	0.98[0.84,1.14]	12
**Hiatt et al.** (1994)	USA	N	238/43 432	0.8[0.5,1.3]	14
**DeAntoni et al.** (1997)	USA	40–95	N/95961	1.07[0.88,1.3]	2
**Lynge et al.** (2002)	Denmark	all years	46/57931	0.98[0.73, 1.31]	12
**Rohrmann et al.** (2005)	USA	> 35	78/3373	2.03[1.24, 3.32]	8
**Romero et al.** (2012)	Brazil	> 40	58/2118	0.23[0.03, 1.70]	5
**Siddiqui et al.** (2014)	USA	40–75	6023/49405	1.10[1.04, 1.17]	24

Note: *: undefined.
